# Effectiveness of Action in India to Reduce Exposure of *Gyps* Vultures to the Toxic Veterinary Drug Diclofenac

**DOI:** 10.1371/journal.pone.0019069

**Published:** 2011-05-11

**Authors:** Richard Cuthbert, Mark A. Taggart, Vibhu Prakash, Mohini Saini, Devendra Swarup, Suchitra Upreti, Rafael Mateo, Soumya Sunder Chakraborty, Parag Deori, Rhys E. Green

**Affiliations:** 1 Royal Society for the Protection of Birds, Sandy, United Kingdom; 2 Instituto de Investigación en Recursos Cinegéticos, IREC (CSIC-UCLM-JCCM), Ciudad Real, Spain; 3 Department of Plant and Soil Science, School of Biological Sciences, University of Aberdeen, Aberdeen, United Kingdom; 4 Bombay Natural History Society, Mumbai, India; 5 Centre for Wildlife Conservation, Management and Disease Surveillance, Indian Veterinary Research Institute, Izatnagar, Uttar Pradesh, India; 6 Conservation Science Group, Department of Zoology, University of Cambridge, Cambridge, United Kingdom; University of Durham, United Kingdom

## Abstract

Contamination of their carrion food supply with the non-steroidal anti-inflammatory drug diclofenac has caused rapid population declines across the Indian subcontinent of three species of *Gyps* vultures endemic to South Asia. The governments of India, Pakistan and Nepal took action in 2006 to prevent the veterinary use of diclofenac on domesticated livestock, the route by which contamination occurs. We analyse data from three surveys of the prevalence and concentration of diclofenac residues in carcasses of domesticated ungulates in India, carried out before and after the implementation of a ban on veterinary use. There was little change in the prevalence and concentration of diclofenac between a survey before the ban and one conducted soon after its implementation, with the percentage of carcasses containing diclofenac in these surveys estimated at 10.8 and 10.7%, respectively. However, both the prevalence and concentration of diclofenac had fallen markedly 7–31 months after the implementation of the ban, with the true prevalence in this third survey estimated at 6.5%. Modelling of the impact of this reduction in diclofenac on the expected rate of decline of the oriental white-backed vulture (*Gyps bengalensis*) in India indicates that the decline rate has decreased to 40% of the rate before the ban, but is still likely to be rapid (about 18% year^−1^). Hence, further efforts to remove diclofenac from vulture food are still needed if the future recovery or successful reintroduction of vultures is to be feasible.

## Introduction

Three species of vultures endemic to South Asia, oriental white-backed vulture (*Gyps bengalensis*), long-billed vulture (*G. indicus*) and slender-billed vulture (*G. tenuirostris*), are listed as being threatened with extinction after rapid population declines in the Indian subcontinent, which began in the 1990s [1], [2], [3]. The oriental white-backed vulture population in India in 2007 was estimated at one-thousandth of its level in the early 1990s [3]. Veterinary use of the non-steroidal anti-inflammatory drug (NSAID) diclofenac is the major cause of these declines [4], [5], [6], [7]. Diclofenac has been used to treat symptoms of disease and injury in domesticated ungulates in many parts of the subcontinent since the 1990s [8]. The effects of diclofenac on captive oriental white-backed vulture, African white-backed vulture (*G. africanus*), Cape griffon vulture (*G. coprotheres*) and Eurasian griffon vulture (*G. fulvus*) have been studied experimentally. In all species, death occurred within a few days and extensive visceral gout and kidney damage were observed post mortem [4], [9], [10]. Vultures that died in these experiments showed similar pathology to that found in the majority of vulture carcasses collected from the wild since declines began [4], [5], [6], [9], [11]. A large-scale survey of the amount of diclofenac in liver tissue from carcases of domesticated ungulates available to vultures as food in India in 2004–2005 showed that the prevalence and concentration of the drug was sufficient to cause the observed rapid population declines [7], [12]. Approximately 10% of carcasses were found to have detectable levels of diclofenac [12].

After research had indicated the adverse effects of diclofenac on vultures, the governments of India, Pakistan and Nepal commenced actions to prevent the contamination of vulture food supplies with the drug [13]. India's National Board for Wildlife recommended a ban on veterinary use on 17 March 2005. In May 2006, a directive from the Drug Controller General of India was circulated to relevant officials, requiring the withdrawal of manufacturing licences for veterinary formulations of diclofenac. This directive was further strengthened in 2008, when it was made an imprisonable offence to manufacture, retail or use diclofenac for veterinary purposes.

In this paper, we analyse data from three surveys of diclofenac concentrations in liver samples from carcasses of domesticated ungulates in India before and after the ban [12], [14] in order to estimate the change in the expected rate of mortality caused by diclofenac in oriental white-backed vultures and the expected trend in their population. Our analysis is restricted to oriental white-backed vulture because this is the only species for which the relationship between dose and mortality has been measured [4], [9]. However, we expect that our conclusions concerning this species will also be relevant to the conservation of the two other threatened *Gyps* species in South Asia.

## Methods

### Field sampling

Liver samples were collected from carcasses of domesticated ungulates during three survey periods: T1  =  May 2004–July 2005, T2  =  April–December 2006, T3  =  January 2007–December 2008. Samples were collected from carcasses deposited at carcass dumps managed by local government corporations, co-operatives, private companies and individuals, and cattle welfare charities. Sampling locations were typical of sites formerly used by large numbers of foraging *Gyps* vultures. Samples were also collected from slaughterhouses during T1 (15% of samples), but not during subsequent surveys. Protocols for sample collection and storage have been reported previously [12], [14].

GPS co-ordinates of sample collection sites were recorded. Each site in the T2 and T3 surveys was assigned to one of 21 site clusters previously identified during an analysis of the T1 survey data [7]. Site-cluster assignment was based upon the site being nearer to the geodesic centroid of a particular cluster than to that of any other cluster. Sample sites were always within 186 km of the geodesic centroid of their cluster. Samples were gathered opportunistically when and where it was possible to obtain access and permission to collect. For logistical reasons the geographical distribution of sampling effort differed among the three surveys. Site clusters covered in T2 and T3 were a subset of those covered during T1. The number of samples taken in each cluster differed among surveys.

### Sample extraction and quantification of diclofenac concentration

Weighed sub-samples of ungulate liver were homogenized in acetonitrile. Diclofenac concentrations in the extracts were determined by liquid chromatography-electrospray ionisation mass spectrometry. The limit of quantification (LOQ) for this technique, back-calculated to the concentration in wet tissue, was 0.01 ppm (0.01 mg kg^−1^). Detailed protocols for sample extraction and diclofenac quantification have been reported previously [12], [14].

### Statistical analysis

The objectives of our analyses were to estimate (1) changes in the level of exposure of vultures to diclofenac over time, (2) consequent changes in the average proportion of oriental white-backed vultures expected to be killed by diclofenac per meal of carrion consumed, and (3) the expected annual rate of decline of a model population of oriental white-backed vultures that would have been stable in the absence of diclofenac. The analysis followed Steps 1–8 of the procedure described in a previous analysis of the T1 survey data [7], except that Step 2 of the procedure was omitted. This was because the previous analysis showed that variation in the measured diclofenac concentration among sub-samples taken from the liver of the same ungulate had a negligible effect on the outcome of the analysis. This source of variation could therefore be ignored. The procedure fits a statistical model to the frequency distribution of the concentrations of diclofenac measured in samples of liver taken from carcasses of domesticated ungulates. It then estimates from the data for liver the distribution of diclofenac concentrations averaged over all edible tissues of the ungulate carcasses and, from that, the distribution of doses of diclofenac per unit vulture body mass ingested by vultures feeding on a mixture of tissues. The expected average proportion of vultures killed per meal is then obtained and this result is used in a simulation model of the vulture population to estimate its expected rate of decline.

Step 1 of the procedure required a more elaborate treatment than that used in the previous analysis because the present study compares data from three surveys rather than reporting just one. This step determines the cumulative distribution function (cdf) V(*d_liver_*) of the concentrations *d_liver_* of diclofenac in ungulate livers. For the purpose of the present analysis, it is necessary to determine V(*d_liver_*) for each survey period (T1, T2 and T3), whilst avoiding, as far as possible, the potential bias introduced by differences among surveys in the geographical distribution of sampling sites. In previous analysis of the T1 data [7], V(*d_liver_*) was assumed to be 1 + *f* (U(*d_liver_*) – 1), where *f* is the true prevalence of diclofenac, i.e., the proportion of livers that contained residues of the drug, and U(*d_liver_*) is the cdf of diclofenac concentrations in samples that contained the drug. A proportion of the livers sampled (1 - *f*) have no trace of the drug. In previous analysis [7], a third order complementary log-log distribution was used for U(*d_liver_*) because this distribution gave a good fit to the data. However, this distribution requires the estimation of four parameters, in addition to *f*. To reduce the number of fitted parameters required to describe the diclofenac distributions for the three survey periods, we instead assumed that U(*d_liver_*) was a Weibull distribution, which is determined by just two parameters; a scale parameter *a* and a shape parameter *b*. Using this formulation, U(*d_liver_*)  =  1 - exp(-*a d_liver_^b^*) and V(*d_liver_*) = 1 – *f* exp(-*a d_liver_^b^*). To check whether simplifying the model in this way resulted in an appreciably poorer fit, we compared the Weibull and third order complementary log-log distributions when fitted to the data for samples with detectable diclofenac levels from each of the three surveys. We fitted truncated distributions using a maximum-likelihood method [15], left-censored at the LOQ. We assessed the fit of each distribution using a Kolmogorov-Smirnov one sample test [16]. As expected, because of its larger number of fitted parameters, the third order complementary log-log (CL-L3) distribution gave a better fit than the Weibull for all three surveys. Kolmogorov-Smirnov's D values for CL-L3 vs Weibull for T1, T2 and T3 were 0.042 vs 0.049, 0.030 vs 0.035, 0.043 vs 0.048 respectively, but these differences are small and the fit of all models was good. The significance of the Kolmogorov-Smirnov test was *P*>0.40 in all cases. Hence, we concluded that the Weibull gave an adequate fit, thus allowing a reduction in the number of fitted parameters required and a simplification of subsequent analysis.

**Figure 1:**
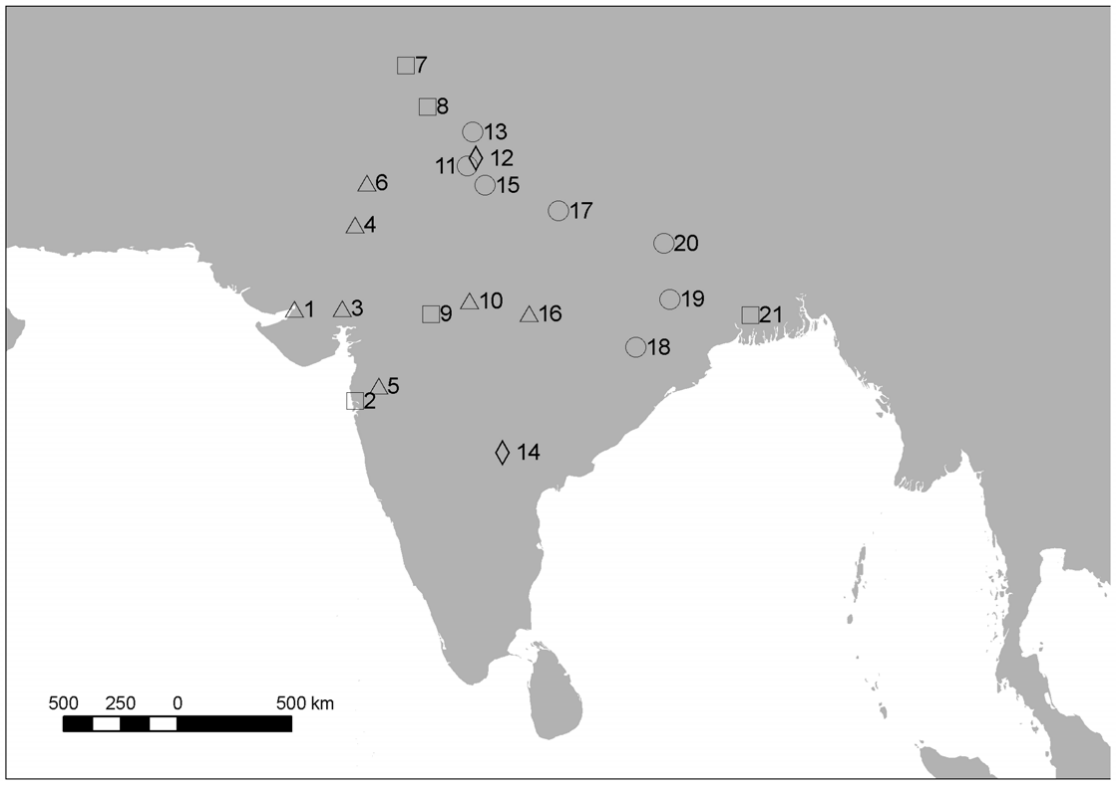
Locations of sampling site clusters in India. The map shows centroids of 21 site clusters at which liver samples were obtained from carcasses of domesticated ungulates. Numbers next to the symbols identify site clusters listed in Table 1. Triangles show clusters sampled in all three surveys (T1, T2, T3), squares show clusters sampled in T1 and T2, diamonds, T1 and T3, and circles T1 only.

If the number and thus the proportion of samples taken within each site cluster had remained the same for all three surveys, it would have been acceptable to estimate *f*, *a* and *b* separately for each survey and then make a direct comparison of these estimates. However, given that some of the site clusters sampled in T1 were not sampled in T2 or T3 or both, and that different numbers of samples were taken within clusters that were sampled in more than one survey, we considered it necessary to model the prevalence and concentration of diclofenac as varying with site cluster (S) and time period (T) (see Table 1 and Figure 1 for the distribution of samples in surveys T1 to T3). Site cluster and survey period were treated as factors in the Weibull models. This allowed us to simulate the prevalence and concentration of diclofenac that would be expected if the geographical distribution of sampling had actually been the same in all three surveys. Since models with site-cluster effects were only used to estimate changes over time, we reduced the number of fitted parameters required by combining data for the seven site clusters that were only sampled in the T1 survey and treating these as if they came from just one cluster in all analyses. We used different approaches to represent site-cluster and survey-period variation in the parameters for the prevalence and concentration components of the model. For true prevalence *f*, which is a proportion, we assumed that the odds of a sample containing diclofenac were the product of two constants; a site-cluster effect *g* and a survey-period effect *h*. Hence, for the *i*th site cluster and the *j*th survey period, *f_ij_*/(1 – *f_ij_*), the logit transformation of *f_ij_* was assumed to be given by the product *g_i_ h_j_*. The scale and shape parameters *a* and *b* of the Weibull model were assumed to be products of site-cluster (*m* and *q*) and survey-period (*n* and *r*) effects so that *a_ij_* = *m_i_ n_j_* and *b_ij_* = *q_i_ r_j_*. The *g*, *m* and *q* factors had a number of levels equal to the number of site clusters, except that the site clusters only sampled in T1 were pooled (as explained above). The *h*, *n* and *r* factors each had three levels, one for each survey period, but the parameter values for the first period, *h_1_*, *n_1_* and *r_1_* were fixed at 1, so there were two parameters to be estimated for each factor. We call these the S+T formulations of the model for true prevalence, scale parameter and shape parameter.

**Table 1:** Numbers of ungulate liver samples collected in each of 21 site clusters in three survey periods: T1  =  May 2004–July 2005, T2  =  April–December 2006, T3  =  January 2007–December 2008.

	Number of liver samples		
Cluster	T1	T2	T3
1	28	164	85
2	163	200	0
3	38	58	74
4	159	152	151
5	26	41	262
6	150	187	152
7	90	169	0
8	63	171	0
9	83	110	0
10	92	106	236
11	59	0	0
12	134	0	127
13	150	0	0
14	161	0	143
15	42	0	0
16	25	20	21
17	64	0	0
18	52	0	0
19	54	0	0
20	121	0	0
21	94	110	0
Total samples	1848	1488	1251
Samples with diclofenac	186	165	70
Percentage with diclofenac	10.1	11.1	5.6
Mean concentration (ppm)	0.994	0.874	0.569

	Percentage of samples		
Species	T1	T2	T3
Camel	0.1	0.0	0.2
Cattle	48.3	63.8	60.1
Water buffalo	46.6	29.7	34.9
Sheep	2.6	2.6	2.4
Goat	0.2	3.6	2.2
Horse	2.3	0.3	0.2
Unidentified	0.0	0.1	0.0

Also shown are the total numbers of samples taken, the number and proportion of them in which diclofenac was detected, the arithmetic mean concentration of diclofenac (ppm wet weight) in the samples in which the compound was detected and the species composition of the ungulates from which liver tissue was sampled.

Our eventual goal was to fit a single statistical model to the data from all three surveys with appropriate S+T formulations for the parameters that determined prevalence and concentration. However, we first conducted preliminary analyses separately for (1) the prevalence and (2) the concentration components of the model to compare the effects on model fit of the S+T formulations of the different model parameters and other plausible model formulations. In the analyses of prevalence, this was done by fitting logistic regression models to the data on apparent prevalence. Apparent prevalence was the proportion of samples with diclofenac concentrations above the LOQ, not including the undetected contaminated samples with levels of diclofenac below the LOQ. A logistic regression model with the presence/absence of detectable diclofenac as the binary dependent variable and the additive main effects of site cluster and survey period included as factors is equivalent to the S+T formulation of the model of true prevalence described above. In separate analyses of diclofenac concentrations, we fitted a truncated Weibull distribution of concentrations, left-censored at the LOQ, using a maximum-likelihood method [15]. This analysis only used data for samples with detectable diclofenac. For both the logistic regression analyses of apparent prevalence and the truncated Weibull models of concentration, plausible alternative models of apparent prevalence and concentration with various formulations were fitted and compared by calculating their Akaike Information Criterion (AIC) values.

After completing the preliminary analyses of apparent prevalence and concentration, a combined Weibull model including both components, with the selected S+T formulation, was fitted to the full dataset using a maximum-likelihood method [15]. Confidence limits for the parameters of the model were obtained by taking 10,000 bootstrap samples of the data, with bootstrapping being performed by site cluster. The model was then fitted to each bootstrap sample and the central 9,500 estimates of each parameter were taken to be the 95% confidence limits.

We estimated the impact of the observed level of diclofenac contamination on the proportion of oriental white-backed vultures killed by diclofenac per meal using Steps 3 – 7 of the procedure developed previously [7]. Estimates of the parameters required for the calculations were taken from this earlier analysis. The procedure requires the following assumptions. (a) Vultures eat a meals of ungulate tissue of uniform size at intervals *F* of either two or three days, such that that their energetic requirements are met. (b) The concentration of diclofenac in each meal is that found in all edible tissues of the ungulate combined, which is proportional to the diclofenac concentration in the animal's liver, as determined previously [7]. (c) The distribution of diclofenac concentrations in meals is given by the product of the ratio of concentration in the whole carcase to that in the liver and the distribution of liver concentrations fitted to the results of the survey of ungulate carcasses described above. (d) The proportion of vultures killed by a given dose of diclofenac is specified by a relationship fitted to data from a dosing experiment conducted previously on captive oriental white-backed vultures [4], [9]. We used a version of this dose-response curve that was fitted after excluding an outlier (Vulture 11) [4], [9], since inclusion of this datum leads to unrealistically high estimates of the rate of population decline [7]. The average proportion of vultures killed per meal, averaged across all meals taken by the vulture population, was then obtained from the probability density function of the dose of diclofenac per unit vulture body weight per meal and the dose-response relationship between diclofenac dose and the proportion of vultures killed. Integration under the curve given by the product of these two functions gives the average proportion of vultures killed per meal [7].

The final step in the procedure (Step 8) [7] used the death rate per meal, as calculated above, in a simple model of the vulture population [5] to estimate the population's expected rate of decline. The model assumes that the population would have demographic rates such that it is stable in the absence of diclofenac, and that the annual adult survival rate *S_0_* is either 0.90 or 0.97. These survival values were considered to span the plausible range in a previous modelling study [5]. The interval between meals *F* is assumed to be either 2 or 3 days. Other details of the model have been described previously [5]. Confidence limits for death rate per meal and population decline rate were obtained using sets of 10,000 bootstrap and Monte Carlo parameter estimates as described previously [7].

## Results

### Differences among surveys in diclofenac prevalence and concentration

**Figure 2:**
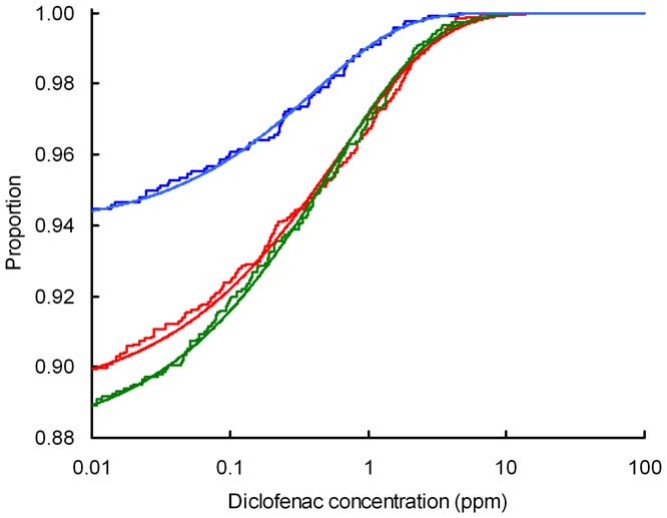
Comparison of the distributions of diclofenac concentrations before and after the ban on the veterinary use of diclofenac. Cumulative distributions of diclofenac concentration (ppm wet weight) in ungulate liver samples from three surveys: red = T1, pre-ban, green = T2, soon after the ban, blue = T3, 7–31 months after the ban are shown by the stepped lines. The curves show cumulative Weibull distributions fitted separately to the data for each survey. Fitted values of prevalence *f,* the scale *a* and shape *b* parameters respectively were T1, 0.110, 1.336 and 0.592; T2, 0.122, 1.458 and 0.597; T3, 0.061, 1.844 and 0.673.

Liver samples were taken from a large number of sites distributed across the northern half of India (Table 1, Figure 1), and came predominantly from carcasses of cattle (*Bos indicus, B. taurus* and hybrids) and water buffalo (*Bubalus bubalis*). The proportion of samples with detectable diclofenac (apparent prevalence), the cumulative distribution of diclofenac concentrations and the arithmetic mean concentration in those samples with detectable levels were broadly similar between the two surveys conducted before and just after the implementation of the ban on diclofenac use for veterinary purposes (T2 cf. T1, Table 1). However, apparent prevalence and mean concentration were both substantially lower in the third survey than in the previous two (T3 cf. T2 and T1, Table 1, Figure 2).

The geographical distribution of samples differed among the three surveys (Table 1, Figure 1). Only seven of the 21 site clusters were sampled in all three surveys. Five site clusters were sampled in T1 and T2, but not in T3, two site clusters were sampled in T1 and T3, but not in T2, and seven site clusters were only sampled in T1 (Table 1). A higher proportion of the site clusters located in western India were sampled in more than one survey than was the case for clusters in the east (Figure 1).

Differences in the distribution of sampling sites among surveys might lead to spurious differences in prevalence or the distribution of concentrations of diclofenac if (a) these varied consistently with location, and (b) site clusters covered in surveys T2 and T3 differed in prevalence or concentration distribution from the site clusters not covered in the later surveys, or covered to a lesser extent. A previous analysis of data from T1 has already revealed significant geographical variation in apparent prevalence [12]. Comparison of apparent prevalence between pairs of survey periods indicated that significant positive correlations also existed across site clusters. Clusters with higher than average apparent prevalence in one survey also tended to have high prevalence in other surveys (Spearman correlation coefficients [16]: T1 vs T2, *r_S_* = 0.504, one-tailed *P* = 0.05; T1 vs T3, *r_S_* = 0.483, one-tailed *P* = 0.10; T2 vs T3, *r_S_* = 0.714, one-tailed *P* = 0.05). Hence, apparent prevalence not only varied geographically, but the pattern of variation among site clusters tended to be consistent through time. However, the equivalent correlation analyses for mean diclofenac concentration in those samples with detectable levels gave no indication that mean concentrations varied consistently among site clusters in different time periods (Spearman correlation coefficients: T1 vs T2, *r_S_* = −0.067; T1 vs T3, *r_S_* = 0.321, one-tailed *P* = 0.25; T2 vs T3, *r_S_* = 0.143, one-tailed *P*>0.25).

Although differences among surveys in the geographical distribution of sampling sites were present and might cause spurious differences in estimates of diclofenac prevalence and concentration distribution, simple non-parametric analyses indicated that differences between surveys remained even after site-cluster differences had been allowed for. A Wilcoxon signed ranks test [16] on differences between pairs of apparent prevalence values for the same cluster during different time periods indicated that apparent prevalence was significantly lower in T3 than in T1 (*T^+^* = 39, *N* = 9, one-tailed *P* = 0.021). However, there was no significant difference for comparisons among the other survey pairs (T2 vs T1, *T^+^* = 43, *N* = 12, one-tailed *P* = 0.396; T3 vs T2, *T^+^* = 19, *N* = 7, one-tailed *P* = 0.234). The equivalent analyses for mean diclofenac concentrations in those samples with detectable levels indicated that concentrations were also lower in T3 than in T1 (*T^+^* = 27, *N* = 7, one-tailed *P* = 0.016), and had a marginally significant tendency to be lower in T3 than T2 (*T^+^* = 18, *N* = 6, one-tailed *P* = 0.078). There was no significant difference between mean concentrations in T2 and T1 (*T^+^* = 35, *N* = 10, one-tailed *P* = 0.246).

These analyses suggest that both the apparent prevalence of diclofenac and its concentration were lower in T3 than in the previous two surveys. However, they also indicate that there was a consistent geographical variation in prevalence, so quantification of the changes requires more elaborate modelling to adjust for differences among surveys in the geographical distribution of sampling.

### Site-cluster and time-period effects on apparent prevalence and diclofenac concentration

Adjustments for possible biases caused by differences among surveys in the geographical distribution of sampling required models of the prevalence and concentration of diclofenac that had independent main effects of site cluster (S) and time period (T). We call these S+T models and described the way we used them in the Methods section. We performed two preliminary analyses, separately for data for apparent prevalence and concentration, to see how the fit of the S+T models compared with that of other plausible model formulations.

Comparisons of AIC values among logistic regression models of apparent prevalence in relation to site cluster and time period indicated that those models with the effects of S and T included on their own fitted the data substantially better than did the null model (Table 2; Models B and C cf. Model A). The effect upon AIC of site cluster was considerably larger than that of time period. However, a model in which the odds of a sample having detectable diclofenac were given by the product of a site-cluster effect and a survey-period effect (S+T) had a considerably lower AIC than either of the single factor models (Table 2; Model D cf. Models B and C). A full model, with proportions specific to each site-time combination (S.T), gave an even lower AIC value, but the AIC difference between this and the S+T model was modest, indicating that the S+T model (Model D) provides an adequate description of the data on apparent prevalence.

**Table 2:** Comparisons between the residual deviance and Akaike Information Criterion (AIC) of various logistic regression models of the variation among site clusters (S) and survey time periods (T) in the apparent prevalence of diclofenac (the proportion of liver samples with detectable levels of the drug).

Model	Model specification	Residual deviance	Number of parameters	AIC	ΔAIC
A	C	184.39	1	186.39	114.39
B	T	154.45	3	160.45	88.45
C	S	53.84	15	83.84	11.84
D	S+T	40.81	17	74.81	2.81
E	S.T	0.00	36	72.00	0.00

A null model in which the proportion was assumed to be constant (C) across site clusters and time periods was compared with models in which the odds of a sample having detectable diclofenac varied either among site clusters or time periods or was given by the product of a site-cluster effect and a time-period effect (denoted S+T). A full model with proportions specific to each site-time combination is denoted S.T.

Comparisons of AIC values were made among truncated Weibull distribution models of the distributions of diclofenac concentrations in those samples with detectable levels (Table 3). In this case, it was possible to formulate the model so that the scale parameter *a* and the shape parameter *b* were separately or both affected by S and T. A model in which the scale parameter varied with time period but not site cluster, and in which the shape parameter did not vary with S or T, gave the lowest AIC value of all models considered (Table 3; Model 2). However, there was only a small difference in AIC between this model and one with an S+T formulation of the scale parameter and a constant shape parameter (Table 3; Model 10). Other models which had the shape parameter as well as the scale parameter dependent on S and/or T did not give a substantial reduction in AIC compared with Model 10. Given that an S+T formulation is necessary to allow adjustment for possible bias caused by differences among surveys in the geographical distribution of sampling, we concluded that the S+T model (Model 10) of concentration fits sufficiently well to be used for this purpose. Hence, we decided that the combined model of prevalence and concentration should have the same formulation as that in Model D of apparent prevalence and that in Model 10 of diclofenac concentration.

**Table 3:** Comparisons between the residual deviance and Akaike Information Criterion (AIC) of various Weibull models of the variation among site clusters (S) and survey time periods (T) in the concentration of diclofenac in liver samples with detectable levels.

Model	Model specification*a*	Model specification*b*	Residual deviance	Number of parameters	AIC	ΔAIC
1	C	C	6391.58	2	6395.58	1.42
2	T	C	6386.16	4	6394.16	0.00
3	C	T	6390.24	4	6398.24	4.08
4	T	T	6385.46	6	6397.46	3.30
5	S	C	6366.60	16	6398.60	4.44
6	C	S	6375.62	16	6407.62	13.45
7	S	S	6336.39	30	6396.39	2.23
8	S	T	6365.56	18	6401.56	7.40
9	T	S	6370.11	18	6406.11	11.95
10	S+T	C	6359.26	18	6395.26	1.10
11	C	S+T	6375.01	18	6411.01	16.84
12	S+T	T	6358.27	20	6398.27	4.10
13	T	S+T	6369.89	20	6409.89	15.73
14	S+T	S+T	6331.04	34	6399.04	4.88
15	S.T	T	6347.35	36	6419.35	25.18
16	S.T	S.T	6283.50	66	6415.50	21.34

A null model in which the scale and shape parameters *a* and *b* of the Weibull distribution of concentrations of diclofenac were assumed to be constant (C) across sites and time periods is compared with models in which the scale parameter *a* and/or the shape parameter *b* varied with site cluster or time period or were given by the product of S and T effects (denoted by S+T). The full model with parameters specific to each site cluster and time combination is denoted by S.T.

### Combined Weibull model of diclofenac prevalence and concentration

We fitted a combined model in which both the true prevalence of diclofenac *f* and the scale parameter *a* of the Weibull distribution of diclofenac concentrations had an S+T formulation, whilst the shape parameter *b* of the Weibull distribution was assumed not to vary with S or T. We then used a maximum-likelihood method [15] to estimate the values of the true prevalence *f_1_* and the scale parameter *a_1_* across all clusters using the data for the first survey period (T1) alone. This was done by estimating the values of *f_1_* and *a_1_* whilst ignoring the effects of site cluster, and with the shape parameter *b* fixed at the value obtained from the combined model of the data from all three surveys with an S+T formulation for both prevalence and the scale parameter. The values of the time-period effects on prevalence and on the scale parameter were then taken from the combined analysis (the *h* and *n* effects: see Methods) and used to calculate *f_2_* and *f_3_* and *a_2_* and *a_3_* values for surveys T2 and T3 respectively. The time-period effects *h* on the prevalence parameter were multiplied by *f_1_*/(1 - *f_1_*) and then back-transformed to give *f_2_* and *f_3_*. The time-period effects *n* on the scale parameter were multiplied by *a_1_* to give *a_2_* and *a_3_*. This procedure simulated the results expected if the geographical distribution of samples in T2 and T3 had been the same as that in T1. These time-period specific estimates of *f* and *a*, together with the estimate of the shape parameter *b* (assumed to be common to all three time periods) are shown in Table 4. The arithmetic mean diclofenac concentration, calculated across all samples that contained residues of the drug, including those with concentrations less than the LOQ, was obtained using the *a* and *b* values for each survey period. The adjusted estimates from the combined model showed that the true prevalence of diclofenac was similar in T1 and T2, but that true prevalence in T3 was lower than in T1 and in T2 (Table 4). The ratio of values in T3 to those from the earlier surveys indicated a reduction in the true prevalence of diclofenac in T3 to about 60% of its value in T1 and T2 (Table 5).

**Table 4:** Estimates of the parameters of a model which describes the true prevalence *f* of diclofenac in liver samples taken during three surveys of ungulate carcasses (T1, T2, T3) and the scale *a* and shape *b* parameters of the Weibull distribution of diclofenac concentrations (ppm wet weight).

	T1				T2				T3			
Parameter	Estimate	95% C.L.			Estimate	95% C.L.			Estimate	95% C.L.		
*f*	0.108	0.086	-	0.130	0.107	0.085	-	0.129	0.065	0.021	-	0.101
*a*	1.305	0.700	-	1.951	1.444	0.763	-	2.115	2.071	1.020	-	3.150
*b*	0.630	0.578	-	0.697	0.630	0.578	-	0.697	0.630	0.578	-	0.697
Mean concentration	0.927	0.229	-	1.565	0.789	0.214	-	1.363	0.446	0.082	-	0.800

The value *b* is assumed to be common to all three surveys. Also shown is the arithmetic mean concentration of diclofenac (ppm wet weight) for those samples which contained the compound, calculated from *a* and *b*. Parameter estimates and their bootstrap 95% confidence limits are shown for each of three surveys.

**Table 5:** Estimates of changes between three surveys of ungulate carcasses (T1, T2, T3) in the true prevalence *f* of diclofenac, the arithmetic mean concentration of diclofenac in livers of animals in which it was present (ppm wet weight), the estimated mean percentage of vultures killed by a meal of mixed tissues, and the annual percentage rate of decline of the vulture population.

			T2:T1				T3:T1				T3:T2			
Parameter	*F*	*S_0_*	Ratio	95% C.L.			Ratio	95% C.L.			Ratio	95% C.L.		
*f*	-	-	0.987	0.971	-	1.007	0.598	0.253	-	0.859	0.605	0.252	-	0.874
Mean conc.	-	-	0.851	0.699	-	1.003	0.480	0.360	-	0.601	0.565	0.430	-	0.699
Death rate	2	-	0.803	0.608	-	1.054	0.211	0.050	-	0.407	0.262	0.065	-	0.483
Death rate	3	-	0.828	0.655	-	1.044	0.244	0.073	-	0.443	0.295	0.094	-	0.513
Decline rate	2	0.90	0.906	0.655	-	1.019	0.357	0.057	-	0.884	0.394	0.074	-	0.886
Decline rate	2	0.97	0.903	0.654	-	1.020	0.351	0.057	-	0.879	0.388	0.073	-	0.881
Decline rate	3	0.90	0.924	0.721	-	1.017	0.413	0.089	-	0.869	0.447	0.114	-	0.872
Decline rate	3	0.97	0.921	0.719	-	1.017	0.406	0.087	-	0.863	0.440	0.113	-	0.866

The interval between meals *F* was assumed to be two or three days and annual adult survival in the absence of diclofenac *S_0_* was assumed to be either 0.90 or 0.97. Ratios of parameter estimates and their bootstrap 95% confidence limits are shown for each pairwise comparison of surveys.

**Figure 3:**
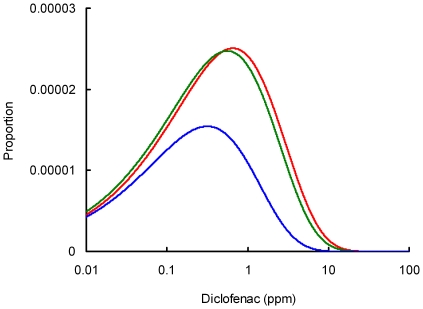
Comparison of probability density functions of diclofenac concentrations in ungulate liver before and after the ban on the veterinary use of diclofenac. Fitted probability density functions are shown of diclofenac concentration (ppm wet weight) in ungulate liver samples from three surveys: red = T1, pre-ban, dark green = T2, soon after the ban, dark blue = T3, 7–31 months after the ban. The curves are derived from a Weibull model in which both the true prevalence of diclofenac *f* (including those with concentrations < LOQ) and the scale parameter *a* of the Weibull distribution of concentrations of diclofenac in those samples are determined by a site-cluster effect and a survey period effect. The shape parameter *b* of the Weibull distribution is assumed not to vary with site-cluster or survey period. Values of *f* and *a* in all three surveys were adjusted so that the results simulate those expected if the 21 site-clusters covered by the T1 (pre-ban) survey had been covered at the same sampling intensity in the second T2 and third T3 surveys.

A similar pattern was seen for the mean concentration of diclofenac in those samples in which the drug was estimated to be present, which was also lower in T3 than in T1 or T2 (Table 4). Calculation of the ratio of values in T3 to those from the earlier surveys indicated a reduction in mean concentration in T3 to 48% and 57% of the values in T1 and T2 respectively (Table 5). Probability density functions (pdfs) calculated from the combined model illustrate this pattern, with the density being lower across all diclofenac concentrations for T3 than for T1 and T2, showing lower prevalence in T3, and the peak of the probability density function occurred at a lower concentration in T3 than in T1 and T2 (Figure 3).

### Proportion of vultures expected to be killed per meal

**Figure 4:**
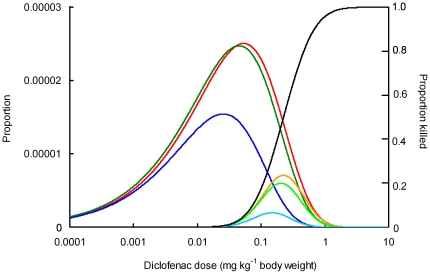
Comparison of probability density functions of diclofenac dose per unit vulture body weight from ungulate tissue before and after the ban on the veterinary use of diclofenac. Probability density functions are shown of estimated diclofenac dose (mg kg^−1^ wet weight) per meal for birds eating a mixture of all edible ungulate tissues and feeding at intervals of three days. Results are shown for three surveys: red = T1, pre-ban, dark green = T2, soon after the ban, dark blue = T3, 7–31 months after the ban. The proportion of vultures expected to be killed by a given dose of diclofenac is shown by the dose-response curve (black, with right-hand y axis). The products of the dose probability density functions and the dose-response curve are shown by the orange, light green and light blue curves for surveys T1, T2 and T3 respectively. The areas under these curves give the estimated proportion of vultures killed per meal.

We estimated the impact of the observed levels of diclofenac contamination on the proportion of oriental white-backed vultures that would be killed per meal using Steps 3 – 7 of the procedure developed earlier for the analysis of the T1 data [7]. The calculation is illustrated in Figure 4, which shows the curve that is obtained by multiplying together the probability density function of the dose of diclofenac per unit vulture body weight per meal and the dose-response relationship between the proportion of vultures killed and the dose of diclofenac ingested. The example shown is for the interval between meals *F* = 3. The integral under this curve gives the proportion of birds killed per meal. For both of the feeding intervals, *F* = 2 and *F* = 3, the death rate per meal was slightly lower in T2 than in T1, but, markedly lower in T3 than in both T2 and T1 (Table 6). The death rate per meal in T3 was 21 – 24% of that in T1 and 26 – 30% of that in T2 (Table 5).

**Table 6:** Estimates of the mean percentage of vultures killed by a meal of mixed tissues assuming that the interval between meals *F* was two or three days, and the annual percentage rate of decline of the vulture population, assuming the two values of *F* and annual adult survival in the absence of diclofenac *S_0_* of either 0.90 or 0.97.

			T1				T2				T3			
Parameter	*F*	*S_0_*	Estimate	95% C.L.			Estimate	95% C.L.			Estimate	95% C.L.		
Death rate	2	-	0.821	0.076	-	3.451	0.660	0.049	-	3.072	0.173	0.004	-	1.231
Death rate	3	-	1.303	0.202	-	4.367	1.080	0.146	-	3.946	0.318	0.016	-	1.649
Decline rate	2	0.90	79.2	14.5	-	99.9	71.8	9.8	-	99.7	28.3	0.9	-	88.4
Decline rate	2	0.97	78.3	14.0	-	99.9	70.7	9.5	-	99.7	27.5	0.9	-	87.9
Decline rate	3	0.90	81.1	23.3	-	99.7	74.9	17.9	-	99.4	33.5	2.1	-	86.2
Decline rate	3	0.97	80.2	22.5	-	99.6	73.8	17.3	-	99.4	32.5	2.0	-	85.7

Parameter estimates and their bootstrap 95% confidence limits are shown for each of three surveys of ungulate carcasses (T1, T2, T3).

### Expected rate of decline of the oriental white-backed vulture population in India

**Figure 5:**
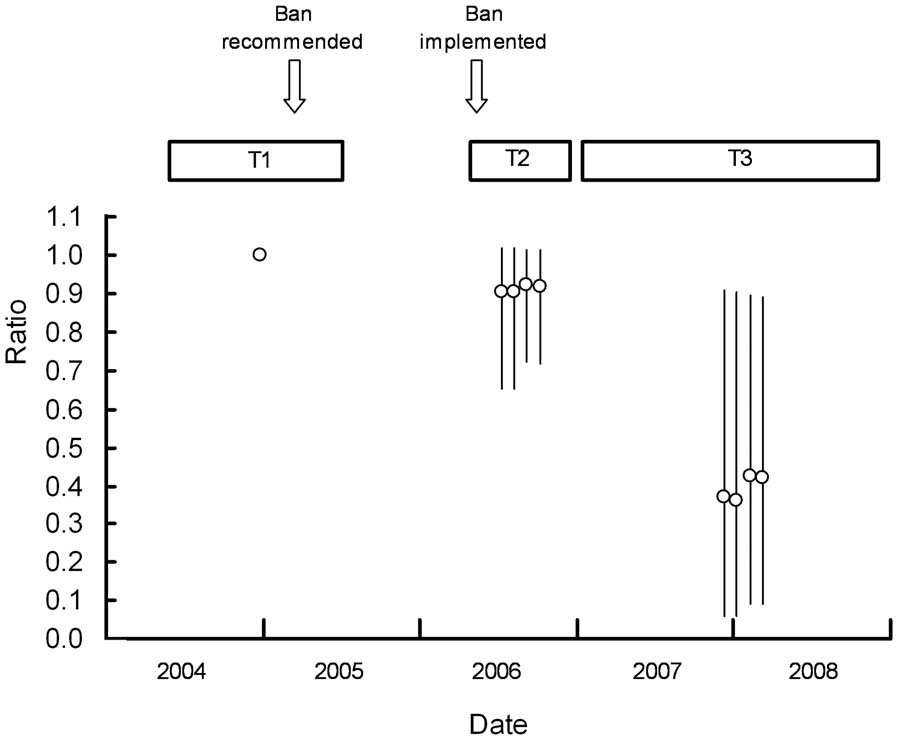
Changes in the expected rate of decline of the oriental white-backed vulture population in India. Circles show the estimated rate of population decline, as a ratio relative to that determined from the T1 survey results in 2004 – 2005. Values are plotted at the mean sampling time for each of the surveys. Horizontal rectangles show the duration of the period covered by the sample collection for each survey. Vertical lines show 95% confidence limits for the ratios. Each of the four adjacent points in a set represents the result for a combination of assumptions (from left to right: *F* = 2, *S_0_* = 0.90; *F* = 2, *S_0_* = 0.97; *F* = 3, *S_0_* = 0.90; *F* = 3, *S_0_* = 0.97). Arrows show the timing of the recommendation by the National Board for Wildlife for a ban on the veterinary use of diclofenac and the withdrawal by the Drug Controller General of manufacturing licences for veterinary formulations of the drug.

The decline rate of the Indian oriental white-backed vulture population, expected from the exposure to diclofenac indicated by survey T1, was 78 – 79% per year with *F* = 2, and 80 – 81% per year with *F* = 3. For both feeding intervals the rate of expected population decline was slightly lower in T2 than in T1, but markedly lower in T3 than in T2 and T1 (Table 6). The decline rate in T3 was 35–41% of that in T1 and 39 - 45% of that in T2 (Table 5). Hence, there was little change in the expected rate of population decline in period T2, immediately after the implementation of the ban on veterinary manufacture of diclofenac in 2006, but by 2007 – 2008 (T3) the expected rate of decline had slowed appreciably (Figure 5).

## Discussion

Our study shows that both the prevalence and concentration of diclofenac in carcasses of domesticated ungulates available as food for vultures in India has fallen markedly since a ban on the veterinary use of diclofenac was implemented. Between the period prior to the ban and the period 7–31 months after the first implementation by the Government of India of measures to prevent the use of diclofenac for veterinary purposes (period T3) decreased by about about half. The estimates of true prevalence, adjusted for samples with low-level contamination below the limit of quantification and for site-cluster effects, were 6.5% in T3, compared with 10.8% and 10.7% in surveys T1 and T2 respectively. Hence, our conclusion about the change in prevalence between surveys holds with or without the statistical adjustments. Similarly, the unadjusted estimates of mean concentration in Table 1 and the adjusted values for mean concentration in Table 4 both show the same pattern of little difference between T1 and T2 but a substantial decline by period T3. We consider that these declines in prevalence and concentration are likely to be representative of the situation in north-western India because of the wide distribution of sampling sites and scale of sampling undertaken (>4,500 carcass samples analysed in T1 – T3). The magnitude of the decline in diclofenac prevalence was probably slightly underestimated because some T1 samples, but no T2 or T3 samples, were taken from slaughterhouses, where diclofenac prevalence was lower than at carcass dumps [12]. However, because only seven of the samples taken at slaughterhouses in the T1 survey were from a site cluster which was sampled in later surveys, this effect is extremely small.

Our estimates of the expected vulture death rate per meal and the expected decline rate of the oriental white-backed vulture population based upon the T1 carcass survey carried out before the ban on diclofenac use was introduced were very similar to those made previously using the same data but with a different method for modelling the distribution of concentrations [7]. This indicates that the changes made in the present analysis to the methods, principally the replacement of the complementary log-log distribution by the Weibull distribution, had a negligible effect on the results.

Based on the data on prevalence and concentration of diclofenac residues presented here and the results of modelling the impact of these residues on the vulture population, the expected rate of decline of the Indian oriental white-backed vulture population has been cut by more than half compared with what it was before the ban. We showed previously that the expected rate of vulture population decline estimated by our method from surveys of diclofenac in ungulate carcasses was higher, though not significantly so, than that observed using repeated counts of vultures during the same period [7]. There are several possible reasons for this difference, if it is real. For example, the restriction of the remaining vultures to areas with lower than average diclofenac prevalence and potential selection for vultures with a higher resistance to the toxic effects of diclofenac [7]. However, despite the possibility of a discrepancy in its absolute level, it seems probable that our conclusion about the decrease in the expected rate of population decline is reliable, because the same assumptions and modelling procedure were used for all three survey periods.

Although the observed reduction in the level of diclofenac contamination of the vulture food supply in India is an encouraging sign of a potential future solution to this urgent conservation problem, it is clear from the continued presence of diclofenac in many carcasses that the problem has not yet been overcome. The most recent estimate of the rate of decline in the oriental white-backed vulture population in India from repeated road transect counts is an annual decline of 44% year^−1^ between 2000 and 2007 [3]. Our ungulate carcase survey results from survey period T3 suggest a recent reduction of the expected rate of decline to less than half of the rate before the ban on diclofenac use. Hence, by scaling down the rate of decline from road transect surveys in 2000–2007 by the ratio of expected decline rates before and after the ban reported in this paper, we estimate an annual decline rate in 2007–2008 at 18% year^−1^. This remains a rapid rate of population decline compared with rates for most other threatened bird populations [17] and one that is unlikely to be fully counteracted by compensatory *in situ* conservation measures such as nest protection and supplementary feeding. Only a very low proportion (< 1%) of ungulate carcasses is required to contain a lethal levels of diclofenac in order to account for the rapid pre-ban population declines of *Gyps* vultures [5], so it may be necessary to remove nearly all diclofenac from the vulture food supply if populations are to recover or be re-introduced successfully from captive-bred stock. There is also a possibility that an Allee effect may occur, caused by reduced social facilitation of foraging at low vulture population densities [18]. This also suggests that almost complete elimination of diclofenac from vulture food may be needed.

Our results indicate that a substantial decrease has occurred in the level of diclofenac contamination in India, but the continued presence of levels of diclofenac lethal to vultures in ungulate carcasses remains a source of major concern. It is now illegal to import, manufacture, retail or use diclofenac for veterinary purposes in India and the continued presence of residues of the drug in ungulate carcasses in 2007–2008 must therefore be caused by illegal veterinary use. Surveys of pharmacy shops in India confirm that, while diclofenac packaged and labelled for veterinary use was rarely offered for sale for use on livestock after the 2006 ban, human formulations of drug were being sold widely for veterinary use in place of veterinary formulations. A similar situation probably exists in Nepal and Pakistan. Pharmacies in India often dispense both human and veterinary medicines, in which case their holding stocks of human diclofenac is not an offence. Dispensing human formulations of diclofenac for use on livestock, together with informal and illegal dispensing of human diclofenac for veterinary purposes by unregistered people, probably accounts for the continued contamination of ungulate carcasses we have observed. Restrictions on the size of vials of injectable human diclofenac to make it less easy to use human formulations on livestock may help to eliminate these illegal practices. A similar situation may also exist in the Punjab province of Pakistan, where diclofenac-caused mortality of oriental white-backed vultures continued after government action to prevent its veterinary use [19]. If the recovery of wild vulture populations is to be achieved, additional efforts are needed to complete the removal of diclofenac from their food supply and to prevent its replacement by other lethal NSAIDs such as ketoprofen [14], [20]. Further effort is also needed to promote the use of the alternative veterinary NSAIDs known not to pose a risk to vultures. At present, the only veterinary NSAID used in India that is known to have low toxicity to vultures is meloxicam [21], [22].
